# Analysis of tympanic sinus shape for purposes of intraoperative hearing monitoring: a microCT study

**DOI:** 10.1007/s00276-021-02859-7

**Published:** 2021-11-24

**Authors:** Małgorzata Bilińska, Tomasz Wojciechowski, Jacek Sokołowski, Kazimierz Niemczyk

**Affiliations:** 1 Department of Dentistry and Oral Health, Aarhus University, Midtjylland, Aarhus, Denmark; 2 Department of Otorhinolaryngology, Head and Neck Surgery, Medical University of Warsaw, Banacha 1a Street, 02-202 Warsaw, Poland; 3 Department of Descriptive and Clinical Anatomy, Medical University of Warsaw, Chałubińskiego 5, 02-004 Warsaw, Poland

**Keywords:** Tympanic sinus, Temporal bone, Electrocochleography, Tympanoplasty, Computed tomography

## Abstract

**Purpose:**

Sinus tympani is the space in the retrotympanum, with variable morphology. Computed tomography is a common tool to investigate sinus tympani anatomy. During cochlear implantation or tympanoplasty, electrocochleography can be used for hearing monitoring. In such a surgical strategy the electrode is placed in the round window’s region throughout posterior tympanotomy. Common accessible needle-shaped electrodes using is difficult in achieving intraoperative stabilization. The aim of the study is to assess the dimensions and shape of sinus tympani, basing on the micro computed tomography scans for purposes of establishing the possible new electrocochleography electrode shape.

**Materials and methods:**

Sixteen fresh frozen cadaveric temporal bones were dissected. MicroCT measurements included the depth and the width of sinus tympani, width of facial canal with stapedius muscle chamber. Obtained data were analyzed statistically with the use of RStudio 1.3.959 software.

**Results:**

The highest average width of sinus tympani amounted for 2.68 mm, depth measured at the round window plane for 3.19 mm. Width of facial canal with stapedius muscle chamber highest average values at the round window plane- 3.32 mm. The lowest average minimum and maximum values were calculated at the 1 mm above the round window plane. The highest average posterior tympanotomy width was 2.91 mm.

**Conclusions:**

The shape of the tympanic sinus is like a trough with the narrowest and deepest dimensions in the middle part. The ST shape and dimensions should be taken into account in constructing the ECochG electrode, designed for optimal placement through posterior tympanotomy approach.

## Introduction

The central structure in the topography of the posterior wall of the tympanic cavity is facial nerve, or rather the corresponding pyramidal ridge. It divides the posterior wall into lateral and medial spaces. The central point (fulcrum) of the posterior wall, is the pyramidal eminence. There are two lateral and medial spaces. The lateral spaces together form the so-called facial recess. The facial recess is limited laterally by the chorda tympani running along the iter chordae posterius, and medially by the facial nerve canal. The lateral spaces are in the shape of an inverted triangle with the apex downward, where chorda tympani branches from the end of the mastoid part of the facial nerve to form chordo-facial angle. Currently, the description of the medial spaces uses a division into two areas: upper and lower retrotympanum. Upper retrotympanum involves posterior tympanic sinus and sinus tympani.

Sinus tympani (ST) is considered to be largest space in the retrotympanum. It is located medially to the pyramidal eminence, stapedius muscle, and facial nerve and laterally to the posterior semicircular canal [2, 15, 26]. The morphology varies among individuals, although factors affecting the variations in size and shape are unclear. The sinus is subject to great variability in posterior extent, in some cases, the dimension may extend posteriorly beyond the limits of the mastoid portion of facial nerve. The depth of sinus tympani is considered to be the most relevant morphological data [17]. Radiological examination, such as computed tomography (CT) and high-resolution computed tomography (HRCT) are commonly used to investigate pathologies of the temporal bone [3, 28, 29]. Although the anatomy of the middle ear was well evaluated in histological studies [26], investigations based on radiological examination are less common to evaluate prevalence of normal anatomical variations [17, 18, 24, 28].

Some researchers have tried to use ECochG as an intraoperative monitoring tool in middle ear surgeries [11, 19]. Hohmann suggested that it might become a promising tool to assess the function of reconstructed ossicular chain [11]. In cases of inner ear fistula, the inadvertent, unintentional, or unplanned opening of the inner ear can result in severe sensorineural hearing loss [27]. The presence of a fistula of the semicircular canals or the cochlea can be encountered during the removal of cholesteatoma and then hearing monitoring may be useful.

There is crucial during surgery to perform measurements in the shortest possible time.

One of the factors extending the duration of tympanoplasty with hearing monitoring is the placement and stabilization of a needle electrode.

For this reason, it seems beneficial to analyze the anatomy of the retrotympanum and then design a stable electrode for ECochG measurements.

## Aim

The aim of the study is to assess the dimensions (width, depth) and shape of sinus tympani, basing on the micro computed tomography (microCT) scans for purposes of establishing size of the possible new ECochG electrode shape.

## Materials and methods

### Ethical statement

This retrospective study was approved by the Ethics Committee of Medical University of Warsaw (Decision Number KB/69/2015), and abides by the 1964 Helsinki Declaration and its later amendments or comparable ethical standards.

### Temporal bones preparation

The research was conducted on 16 fresh frozen cadaveric temporal bones. At first, all specimens were numbered and labeled with a side. Each of them was prepared in a way to fit into the microCT scanner. The sinus tympani, otic capsule with a bony labyrinth were intact in every specimen.

All the scans were obtained with the scanner Phoenix Xray (GE Sensing & Inspection Technologies, Niels-Bohr-Str. 7, 31,515 Wunstorf, Germany) with parameters: voxel dimensions 0.07 × 0.07 × 0.07 mm; the exposition performed with source voltage of 120 kV and current of 70 mA. All scans were analyzed in RadiAnt DICOM Viewer 2020.1 (64-bit) and obtained data were analyzed statistically with the use of RStudio 1.3.959 software.

### Measurements procedure

**Fig. 1:**
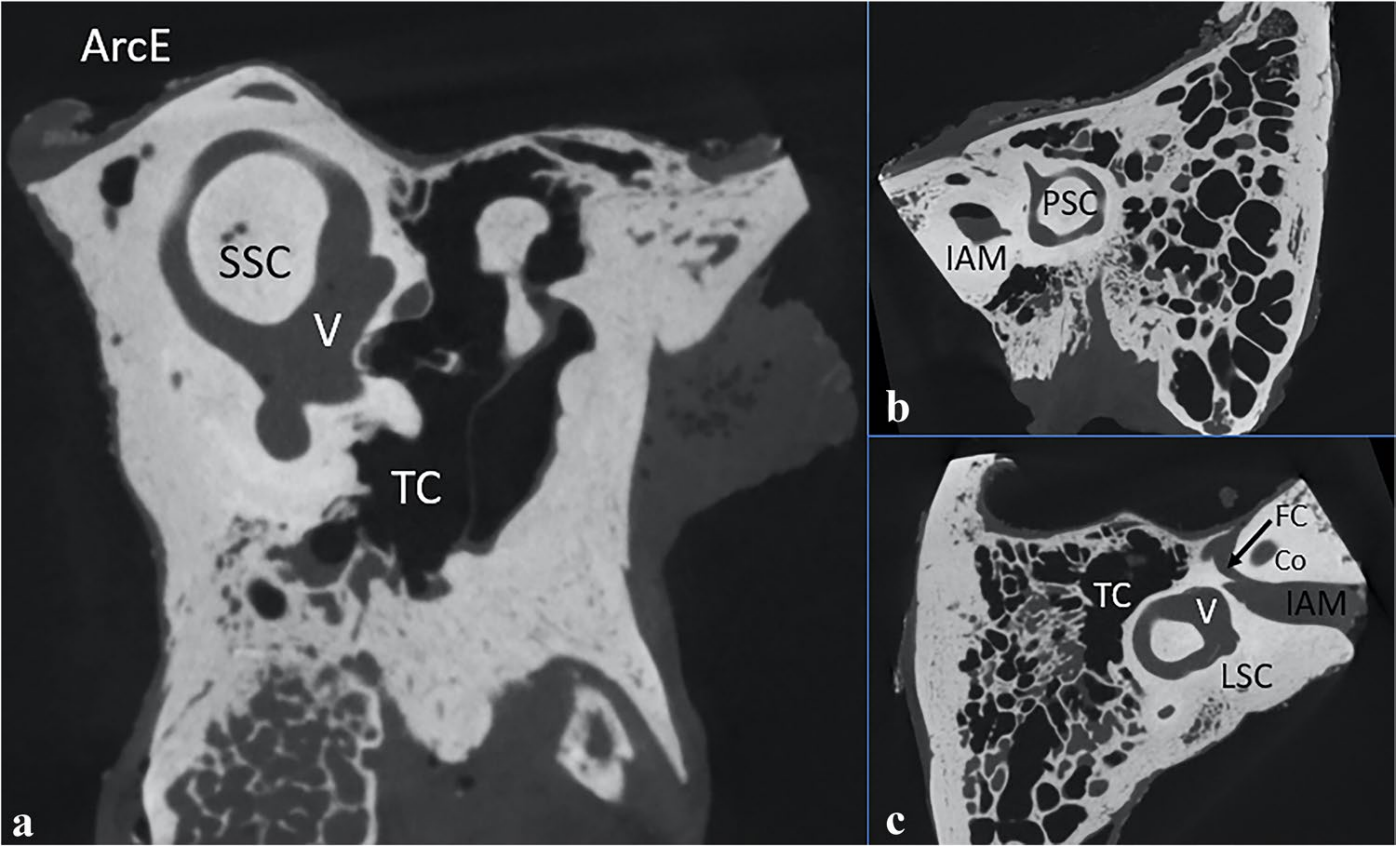
Basic planes used in the study for measuring purposes. **a** Pöschl plane (anterior semicircular canal); **b** posterior semicircular canal plane; **c** lateral semicircular canal plane; *SSC* superior semicircular canal, *ArcE* arcuate eminence, *TC* tympanic cavity, *LSC* lateral semicircular canal, *V* vestibule, *IAM* internal acoustic meatus, *PSC* posterior semicircular canal, *Co* cochlea

In the first phase, the reference planes were established using MultiPlanar Reconstruction (MPR) option to acquire images in the planes of anterior, posterior and lateral semicircular canals (Fig. 1). Table 1 presents variables and symbols analyzed in present study and description how they are defined (Table 2).

**Table 1:** Variables and symbols used in present study

Variable	Description
wST	Width of sinus tympani measured from most anterior point of pyramidal eminence/pyramidal crest to most lateral point of cochlear promontory
dST	Depth of sinus tympani measured from half wST distance to most posterior point of sinus tympani
FN + SM	Width of facial canal and stapedius muscle chamber from most lateral point of facial canal to most medial point of stapedius muscle chamber on the level of the round window
wPT	Width of facial recess (probable posterior tympanotomy) at the level of iter chordae posterius (IChP) opening to tympanic cavity measured from most medial point of IChP opening to most lateral point of facial canal

Variables and symbols used in present study and description how they are defined; − 1/ + 1 added to variable means the level superior or inferior to the round window level

**Table 2:** Statistical analysis of variables

Kruskal–Wallis test			
	FN + SM	wST_RW	dST_RW
*p* value	0.30	0.00001	0.57

Mann–Whitney *U* test			
Compared data/p-value	wST_RW-1	wST_RW	wST_RW + 1
wST_RW-1	x	0.001	0.00006
wST_RW	0.001	x	0.003
wST_RW + 1	0.00006	0.003	x

Kruskall-Wallis test was used to calculate statistically important differences between groups only in width results (*p* value: 0.00001). The Mann–Whitney *U* test confirmed statistically important differences between all groups within width variable

At first, one person has established the protocol and prepared the images. Measurements were performed independently by two researchers, based only on presumed consensus. After analyzing of potential differences reasons, it was found that researchers have chosen slightly different round window measurements planes. When analyzing results the width of the round window over 1 mm has generated a change in the remaining measurement parameters. Therefore it was assumed that the measurement point would be the plane passing through the half of the round window membrane in the plane of the posterior semicircular canal. Repeatability of the method has been obtained in re-measurements in the all assessed parameters. After the correction in the method, there was observed statistically important correlation between measurements of Researcher No 1 and No 2 at the level of round window 1 mm above and one millimeter below in assessment FN + SM-1, dST_RW, dST_RW, wST_RW + 1, wPT.

For the best visualization, the medial retrotympanum requires the use of oblique sagittal plane (Pöschl projection), where temporal bone is viewed from its anteromedial to posterolateral aspects.

Poschl projection is parallel to the superior semicircular canal—it allows the identification of the cochlear promontory, round window and its posterior pillar. This projection is widely used in temporal bone studies [5, 30].

**Fig. 2:**
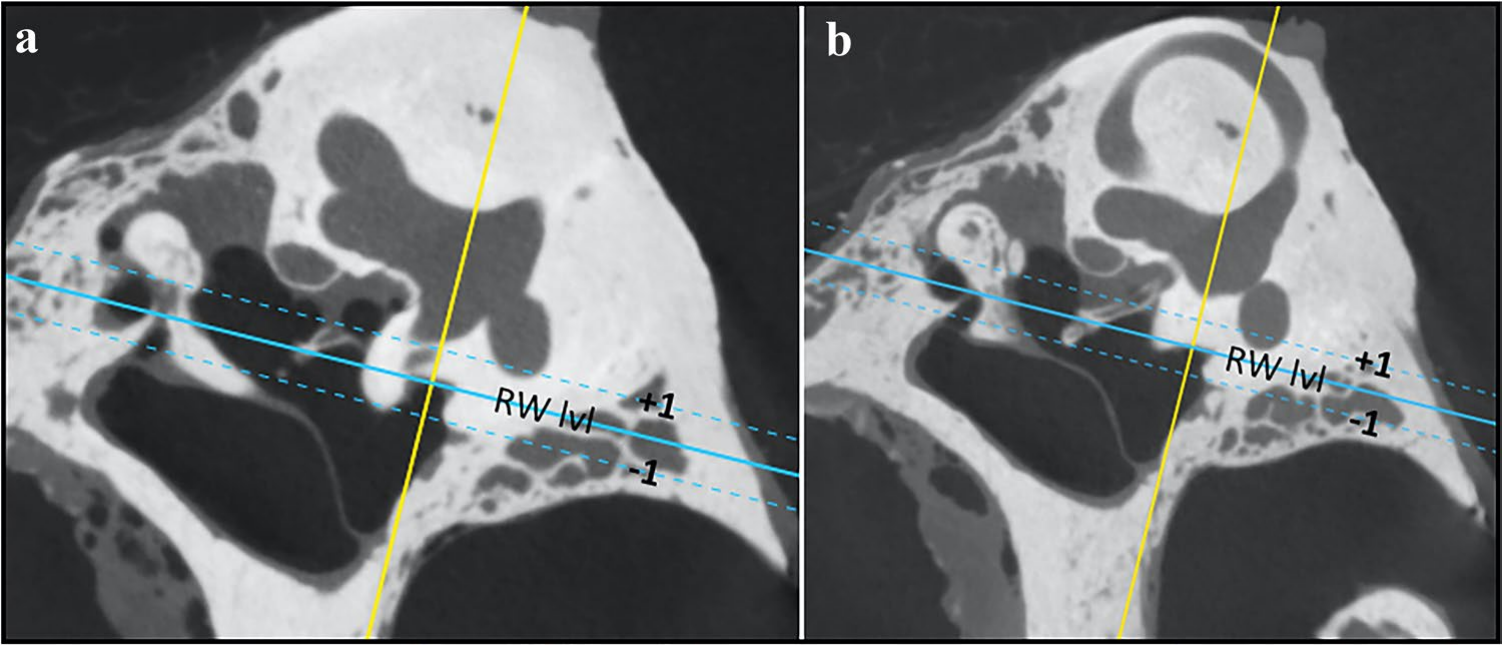
Planes used for measurements in the study **a/b** *Blue line* shows the level of round window (*RW lvl*), *blue dotted lines* show levels 1 mm above (*RW lvl -1*) and 1 mm below round window (*RW lvl -1*), *yellow line* plane paralel to posterior semicircular canal

**Fig. 3:**
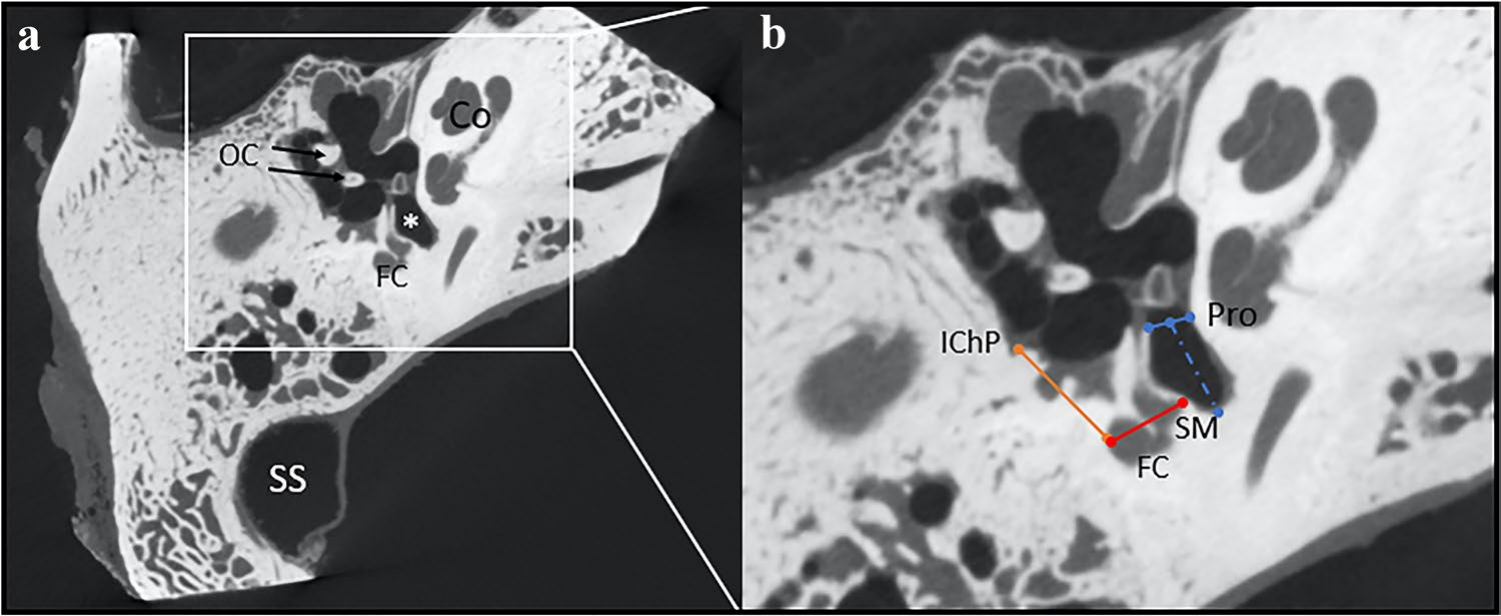
**a** General overview of the measurements area **b** Detailed view shows the distances measured; *OC* ossicular chain, *SS* sigmoid sinus, *Co* cochlea, *FC* facial canal, *white asterisk* sinus tympani, *IChP* iter chordae posterius, *Pro* promontory, *SM* stapedius muscle, *orange line* width of posterior tympanotomy (wPT), *red line* width of facial canal and stapedius muscle chamber (FN + SM), *blue line* width of sinus tympani, *blue dotted line* depth of sinus tympani

**Fig. 4:**
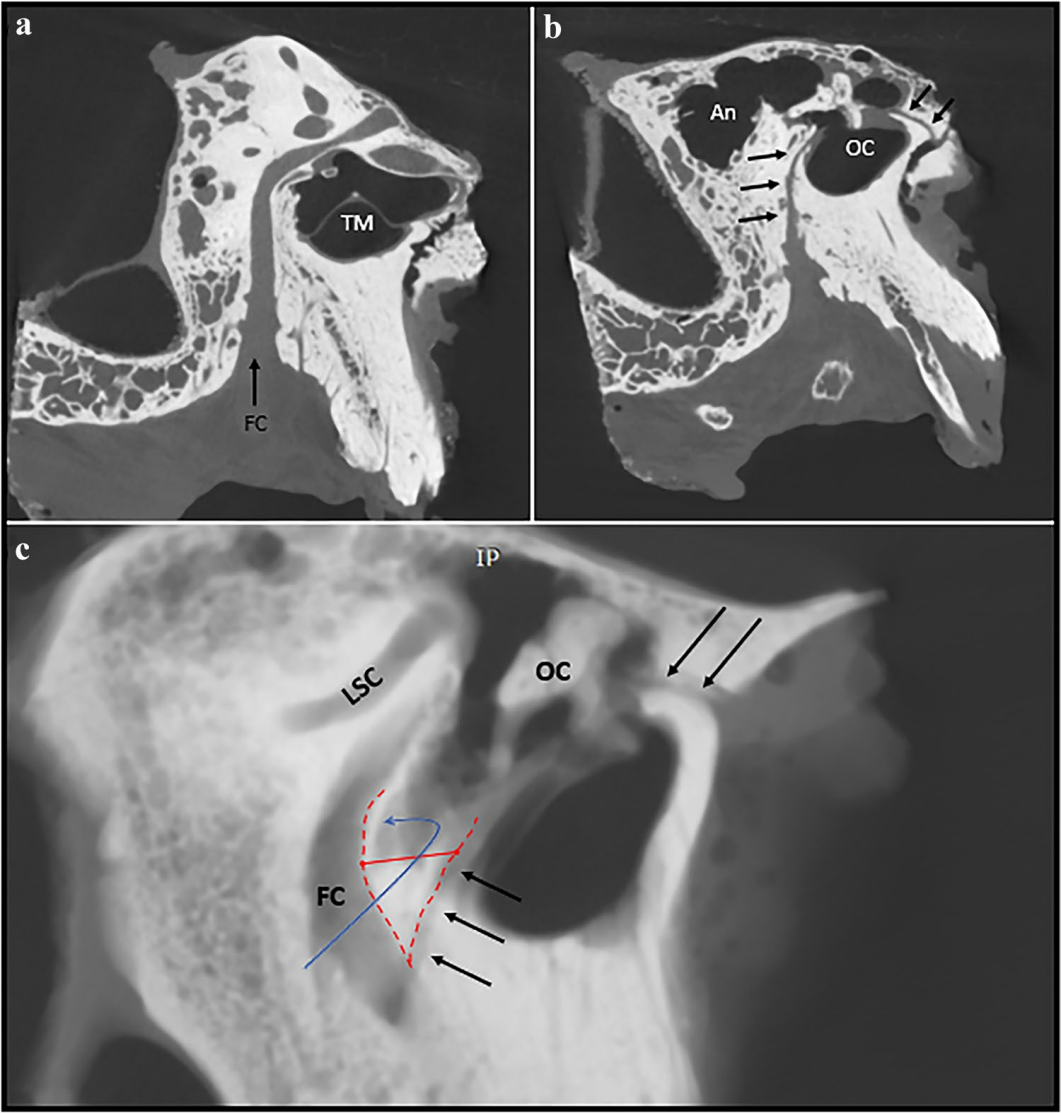
**a** View of facial canal in its plane **b** View of chorda tympani course **c** The route of placement the ball electrode for intraoperative ECochG measurements; *FC* facial canal, *TM* tympanic membrane, *An* mastoid antrum, *OC* ossicular chain, *three black arrows* iter chordae posterius, *two black arrows* iter chordae anterius, *LSC* lateral semicircular canal, *red dotted line* chordo-facial angle – the area of posterior tympanotomy, *red line* width of sinus tympani, *blue line* with arrowhead possible route to sinus tympani through posterior tympanotomy

**Fig. 5:**
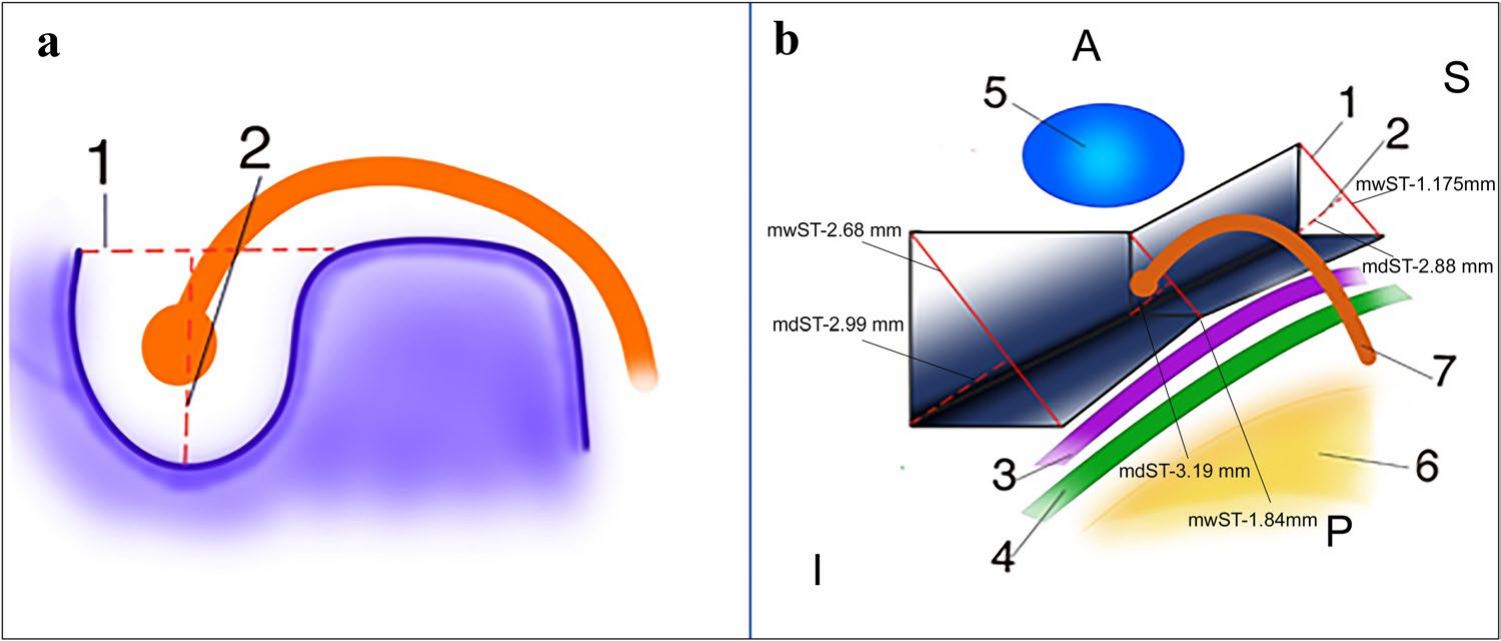
**a** A way to anchor the spherical tip of hook electrode **b** The estimated shape of sinus tympani with its positional relationship with structures in vicinity. Directions: S- superior, I- inferior, A- anterior, P-posterior 1. width of sinus tympani (wPT), 2. depth of sinus tympani(dST), 3. stapedius muscle, 4. facial nerve, 5. round window, 6. bed created during the antromastoidectomy. mwST- mean width value of sinus tympani, mdST- mean depth value of sinus tympani

In the plane parallel to the plane of lateral semicircular canal, three sets of measurements on three different levels were obtained. Set of measurements contained of sinus tympani width (wST), depth (dST) and width of facial canal with stapedius muscle chamber (FN + SM). All these measurements were taken at the level of round window, 1 mm superiorly and 1 mm inferiorly (Fig. 2). Next, in the same plane, at the level of iter chordae posterius opening to the tympanic cavity, width of facial recess (probable posterior tympanotomy) was measured (wPT, Fig. 3). Combined values of wST, dST and wPT may be of help in estimation of probe diameter. The FN + SM value corresponds to possible radius of hook-shaped probe (Figs. 4 and 5). The type of tympanic sinus was assessed according to Marchioni’s classification from shallow through deep to very deep tympanic sinuses [16].

## Results

There was possible to perform measurements of the tympanic sinus based on available micro- Computed Tomography. The CT-scans analysis provided enough information and the anatomical structures such as stapes head, round window, tympanic sinus space were delineated on the CT scan.

### Descriptive statistics

#### Tympanic sinus width

The average width of the tympanic sinus reached the highest values at the level 1 mm below the round window and amounted to 2.68 mm (SD 0.76). The width of the tympanic sinus decreased with the higher position of the measurement plane. At the round window level, it was 1.84 mm (SD 0.61). 1 mm above round window was 1.175 (SD 0.46). A similar trend occurred in the analysis of the maximum and minimum widths of the tympanic sinus.

#### Tympanic sinus depth

The highest average tympanic sinus depth was measured at the round window plane and reached the highest value 3.19 mm (SD 1.03). At the level 1 mm below the round window it was 2.99 mm (SD 1.17) and 1 mm above the round window was 2.88 mm (SD 0.71). The maximum and minimum depth’s values were maintained a similar trend to the average values. There were found four type A tympanic sinuses, ten were classified as type B and two of them—type C, according to Marchioni’s classification.

#### Facial nerve/stapedius muscle width (FN + SM)

The FN + SM achieved the highest average values at the round window plane 3.32 mm (SD 0.60). The lowest average minimum and maximum values were calculated at the 1 mm above the round window plane.

The posterior tympanotomy width—the distance between the facial nerve and chorda tympani.

The highest average posterior tympanotomy width was 2.91 mm (SD 0.67).

#### Data comparing

A statistical comparison of the differences between FN + SM, wST_RW, and dST_RW on three measurements levels. First, in the Kruskal Wallis analysis, statistical differences between parameter levels were calculated. Only in the wST_RW parameter, significant statistical differences were obtained. Then, the Mann–Whitney *U* test was used to calculate the significance levels for each wST_RW level. The lengths of each wST_RW level were statistically significantly different from each other (*p* value < 0.05) (Table 2).

#### The tympanic sinus shape

Based on the collected data, it can be assumed that the shape of the tympanic sinus is like a trough with the narrowest and deepest dimensions in the middle part (Fig. 5).

### Discussion

The variation in the size and shape of the tympanic sinus has been previously investigated in numerous studies.

The surgical access to sinus tympani may be challenging, because of the localization of the facial nerve and the stapedial muscle with its tendon [2].

The hearing improvement is one of the major aim of otologic surgery. Electrocochleography (ECochG) is also an objective method of cochlear function monitoring during near real-time measurements. It allows an indirect assessment of the effectiveness of the ossicular chain reconstruction (by measuring of the cochlea activity) during tympanoplasty.

A method for measuring of the hearing improvement during tympanoplasty has been introduced by Morawski et al. [19]. According to their description, in the first step, canal wall up technique is performed with posterior tympanotomy and ossicular chain reconstruction. In the next stage, earphones are inserted into external auditory meatus and through posterior tympanotomy needle electrode is placed at the nearest point of the round window niche. During measurement, headphones generates acoustic signals at frequencies 500 Hz, 1000 Hz, 2000 Hz, 4000 Hz and intensities that decrease from 90 dB by 10 dB to the hearing threshold. Needle electrode detects electrical responses from the cochlea. Hearing threshold is the lowest sound intensity that generates the electrical response of the cochlea. This method is especially useful in the cases of patients after multiple ossiculoplasties without hearing improvement.

#### Shape

Basing on the measurements in the current study, the tympanic sinus has a trough-shape; the middle part has the narrowest and deepest dimensions. Wang et al. classified ST into three categories: the cup-shaped, the pear-shaped and the boot-shaped, the last one was presented only in patients with congenital aural atresia [29]. In the literature, the shape of sinus tympani was also described as oval [20]

Nitek et al. described in most cases the tympanic sinus as elliptical in shape and its long diameter lies in the vertical plane [21]. Marchioni et al. had proposed three types of ST, that can be detected and classified by HRCT scan: Type A (a limited ST), Type B (a deep ST), and Type C (a deep ST with a posterior extension) [17].

The factors, which affect the variations in shape, volume and the size of the sinus tympani are unclear. The width and depth of the sinus tympani may vary in different anatomic morphology and pneumatization pattern of the temporal bone. In study by Bekci et al., the volume of sinus tympani was significantly greater in patients with pneumatized petrous apex [4]. Our study showed that dimensions of the tympanic sinus were highly variable. Both the width and depth of the sinus tympani were measured in numerous studies and in most of them the measurements are consistent with those in our series [1, 3, 6, 7, 9, 13, 18, 20–23, 26].

#### Width

In the current study, sinus tympani width reached the highest values at the level 1 mm below the round window and amounted to 2.68 mm (SD 0.76) and the values decreased with the higher position of the measurement plane. The results with regard to the width are in consensus with several studies, presented in the Table 3.

**Table 3:** Measurements (width and depth) and shape evaluation of tympanic sinus (ST) in the literature

Author	Year	Sample type	ST width (mm)	ST depth (mm)	ST shape
Abdel Baki et al [1]	2002	Endoscopy	2.4 (1.2–3.3)	2.6 (0.9–6.1)	
Baklaci et al [3]	2019	HRCT	–	1.41 ± 0.68 (0.20–3.00)	
Bekci et al [4]	2020	3D CT	–	–	ST volume rise with petrous bone pneumatization
Cheita et al [6]	2010	Cadaver	2.00 (1.24–2.76)	2.74 (0.5–6.2)	
Chen et al [7]	2005	HRCT	1.98 (± 0.71)	2.25 (± 0.91)	
Donaldson et al [9]	1970	Cadaver	–	3.75	
Kumar et al [13]	2019	Cadaver	1–3 (mean 2.2)	5 (mean 1.72)	
Mazziotti et al [18]	2006	CT	2.50 (1.5- 3.2)	3 (2.3–4.8)	
Niemczyk et al [20]	2003	Cadaver	2.23 (1.0–3.5)		oval
Ozturan et al [22]	1996	Cadaver	1.49 (0.49- 3.87)	2.06 (± 0.60)	
Parlier-Cuau et al [23]	1998	HRCT	–	2.7 (1–9)	
Saito et al. [26]	1971	Cavader	0.96—3.22 (mean 2.14)	2.93 (0.61–5.87)	
Wang et al [29]	2015	3D HRCT	–	–	Cup-shaped, the pear-shaped and the boot-shaped

#### Depth

In the literature, the measurements of the ST depth were provided usually at one level. In the current study, highest average tympanic sinus depth was measured at the level of round window plane (3.19 mm) and the measurement decreased at the level 1 mm below the round window (2.99 mm) and 1 mm above the round window (2.88 mm). The results presented in the literature has shown similar measurements were obtained both from studies based on cadavers, endoscopic and radiologic examination. In the study by Niemczyk et al., the correlation between a deeper tympanic sinus and a more prominent facial canal was observed [20]. In the study by Parlier-Cuau et al., the maximal measurement was 9 mm, according to authors ST remained independent of the mastoid cells. The deepened ST may significantly increase the risk of incomplete excision by the classical transmeatal route [23].

#### Facial nerve/stapedius muscle distance (FN + SM)

According to our knowledge, in the literature there are no descriptions of a specific design of the electrode, which could be used for intraoperative hearing monitoring during middle ear or cochlea surgery through posterior tympanotomy approach [8, 25].

Reported intraoperative ECochG is performed with standard needle electrodes, which does not provide expected stabilization during the procedure. The solution could be designing a ball or needle electrodes, with a shape of the hook, which would allow stable fixation of the electrode in the sinus tympani monitoring [10, 12, 14].

**Fig. 6:**
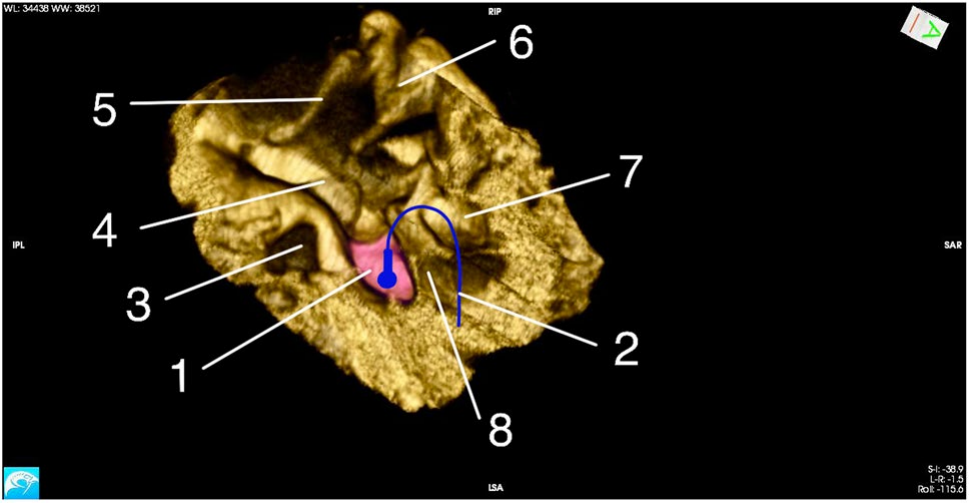
Micro-CT reconstruction presenting sinus tympani with the schematic placement of the ball electrode for electrocochleography. 1. Sinus tympani (pink color) 2. Electrode 3. Round window 4. Stapes 5. Malleus 6. Incus 7. Facial nerve recess 8. Facial nerve and stapedius muscle

The dimensions of ST should be taken into account in constructing the ball electrode, designed for electrocochleography through posterior tympanotomy approach. Combined values of ST width and depth, and the width of posterior tympanotomy may be useful to assess the diameter of the probe, used during electrocochleography. To estimate the size of the ball electrode, the lowest measurement of the width and depth should be assessed. In the planes used for measurements in the current study, the minimal measurements wST ~ 0,5 mm dST ~ 1,1 mm. The distance between 1 mm below the round window and 1 mm above the round window is considered to be the range of insertion of the electrode. Therefore, the estimated height of ST was 2 mm. The FN + SM value corresponds to possible radius of hook-shaped probe, which may be used in intraoperative hearing monitoring. The measurements may be used to design and produce a custom form of the electrode, applied in ear and cochlear surgery (Fig. 6).

The hook-shaped electrode is designed due to the conformation of the retrotympanum and operative approach. The length of the ascending and descending parts of the hook results from the depth of the tympanic sinus and the shape of the bed created during the antromastoidectomy. The shape and size of the rounded center portion of the hook are due to access through the posterior tympanotomy approach in that the length of the FN / SM is the width of the access window. It should be underlined, that during the posterior tympanotomy, both the facial nerve and the stapedius muscles are kept intact.

The electrode is inserted through the posterior tympanotomy similar to a needle electrode. Then it is stabilized on the "bulging" of the facial nerve (pyramidal eminence/pyramidal ridge) and the stapedius muscle at the level of the round window. The spherical tip of the electrode prevents damage of the tympanic mucosa, reducing the probability of bleeding, and increasing the probability of a correct ECochG measurements as it has greater surface of contact than the needle.

## Conclusions

1. Evaluated morphology of sinus tympani revealed the shape of a trough with the narrowest and deepest dimensions in the middle part of the round window. The values increase below and above the level of round window.
2. Minimal measurements of sinus tympani depth and width are 1.01 mm and 1.84 mm. The dimensions should be taken into account in constructing the ball electrode, designed for electrocochleography through posterior tympanotomy approach.

## Data Availability

Data and materials are available from the authors upon request.

## Code availability

Software applications are used under license.
